# *Aedes ægypti* control in urban areas: A systemic approach to a complex dynamic

**DOI:** 10.1371/journal.pntd.0005632

**Published:** 2017-07-27

**Authors:** Marilia Sá Carvalho, Nildimar Alves Honorio, Leandro Martin Totaro Garcia, Luiz Carlos de Sá Carvalho

**Affiliations:** 1 Programa de Computação Científica, Oswaldo Cruz Foundation, Rio de Janeiro, Brazil; 2 Laboratório de Mosquitos Transmissores de Hematozoários – LATHEMA, Instituto Oswaldo Cruz, Fundação Oswaldo Cruz, Rio de Janeiro, Brazil; 3 Núcleo Operacional Sentinela de Mosquitos Vetores – Nosmove/Fiocruz, Rio de Janeiro, Brazil; 4 Pontifícia Universidade Católica do Rio de Janeiro, Rio de Janeiro, Brazil; University of Washington, UNITED STATES

## Abstract

The available strategy for controlling the diseases transmitted by *Aedes ægypti* (dengue fever, Zika, and chikungunya) relies on continued community participation. Despite slogans emphasizing how easy it should be, no country has achieved it since the seventies. To better investigate potentially sustainable interventions, we developed a systemic model based on a multidisciplinary approach, integrating as deeply as possible specialized knowledge and field experience. The resulting model is composed of 4 external and 8 internal subsystems and 31 relationships, consistent with the literature and checked over multiple iterations with specialists of the many areas. We analyzed the model and the main feedback loops responsible for the system’s stability, searching for possible interventions that could shift the existing balance. We suggest the introduction of 1 more player, the local primary health care structure, with the potential to change the undesired equilibrium. The health agents in the areas are the first to detect disease cases, and they could stimulate individuals to inform about potential mosquitoes’ breeding sites and bring timely information to the vector-control program. Triggering such an action could introduce changes in people's attitude through a positive feedback loop in the desired direction.

## Introduction

Due to the Zika virus epidemic in Brazil, an enormous effort has been set in motion to control *A*. *ægypti*, the main vector of several globally important arboviruses including yellow fever [1], dengue [2], and chikungunya virus [3]. Between January and March 2016, 47,828,849 households were inspected and interventions on breeding sites, such as the use of larvicide on drinkable water containers and removal of millions of potential small containers, were performed [4]. A few months later, Zika infections transmitted by mosquitoes in Florida [5] triggered an “aggressive intervention” to control mosquito populations in the continental United States [6]. The effectiveness of this investment is certainly worthwhile in the short term, but the sustainability in the long term is questionable.

One of the topics discussed at the WHO meeting on the challenges presented by this emerging disease was that “there's no evidence that any recent vector-control interventions, including massive spraying of insecticides, have had any significant effect on dengue transmission” [7]. In the mid-20th century, *A*. *ægypti* was eliminated in most countries of the Americas, with the exception of some Caribbean islands, Florida, and Venezuela. However, by the end of 1970, most countries were reinfested. This elimination program was organized in a military-style vertical structure with specific funding that was totally independent from any other health program and with international subsidies, supplies, and personnel training [8]. Additionally, it was based on intensive use of DDT, which is highly effective and persistent in the environment. None of those characteristics are acceptable nowadays—neither the vertical structure nor the use of chemical control posing a risk to the environment [9].

Currently, most of the available strategies are based on intensive population participation. The slogans range from the Brazilian, “A mosquito is not stronger than an entire country” [10] in a Twitter post by @Zikazero to, “Do your part,” or even, “New Yorkers can protect themselves from mosquito bites,” a campaign launched by the New York government and publicized in subway trains. All are based on a series of very detailed recommendations on how to clean water reservoirs, plant pots, bathrooms, and home rain-drainage systems among others [11]. A few hours every week are necessary to fully comply with them all. In fact, a recently published meta-analysis on *A*. *ægypti* control showed that integrated vector management with community participation as active agents of vector control presented the best results [12]. As long as no other effective measure is available (either a vaccine to protect people or the release of some modified mosquito), community participation is extremely important for the control of *A*. *ægypti* populations.

During the first epidemic wave of microcephaly in the Americas, mobilization of public resources, media, and population was substantial. But now, just 1 year later, other headlines have occupied the media, including the risk of urban yellow fever. The Zika transmission will be endemic with sporadic epidemic years, as happened with dengue fever, chikungunya, and the “historical inability to control *Aedes ægypti”* [13]. The risk of pregnant women getting infected will depend on the accumulation of susceptible population in reproductive age. The mobilization of resources and the population involvement will tend to decrease. It should be noted that despite a few studies evaluating efficacy of vector-control programs presenting positive results as proof of concept, few studies address the long-term maintenance of community involvement [14,15]. A recent systematic review concluded that there is “remarkable paucity of reliable evidence for the effectiveness of any dengue vector control method” [16].

Therefore, considering the available interventions, the sustainability and the duration of such an effort should be a major concern of any strategy. This problem can be described as a complex situation in which several components interact nonlinearly, with feedback routes and various time lags generating an emerging dynamic stability [17]. Moving this system in the desired direction—here defined as *A*. *ægypti* population control—has no simple and obvious solution, or one of the countries in which dengue fever has thrived would have already implemented it [7].

The objective of this proposed systemic model is to build a systems map addressing the complexity of *A*. *ægypti* surveillance and control in urban areas in order to inform government authorities, especially the staff working in the vector-control program structure, on possible integrated and encompassing actions besides the usual focused interventions and media campaigns. To the best of our knowledge, we included all intervening factors suggested by available scientific literature and experts’ experience in the model, integrated as fully as possible, connecting and sometimes challenging the common disciplinary borders. As we will see, our model shows some significant gaps of scientific knowledge at these frontiers.

## Methodology

Considering that any complex system can be described in different ways [18], our choice was defined by the following objective: to inform government authorities on possible interventions able to influence the system in a continuous and effective way in the desired direction, bringing an effect as durable and immediate as possible.

As any complex system is in fact an open system, the boundaries of our model were defined based on the envisioned short- and medium-term interventions. For instance, interventions to increase socioeconomic status would probably change the scenario for mosquitoes control; however, the socioeconomic status subsystem was defined as outside the boundaries of our model.

The proposed system is a general overview, composed of several subsystems. As such, it is the first phase of the systems modeling. It is not a nested combination of smaller pieces but a conceptual map that illuminates the whole picture, avoiding both reductionism and dogmatism, the main traps when dealing with complex situations [19]. Relationships within and across any of the subsystems and the external environment are the drivers that shape the system. Relationships can be unidirectional or bidirectional influences, in some cases measurable in some unit, more frequently a route for information flow, and not quantifiable in any way [20]. The internal subsystems are linked through relationships depicted as black arrows. If the relationship triggers a return, it was drawn as a double arrow, 1 dashed, depicting short-circuit feedback loops. If it is a shared aspect, there is just 1 arrow with heads at both ends. Colors may be used to illuminate different aspects of the model. These relationships at some later and more detailed stage can be modeled as quantitative influences. At the top level we are addressing in this paper, most relationships are information flows, not always quantifiable.

Each construct presented in the model is itself a subsystem that can be unfolded and studied in successive steps. The figure is not accessory but central to the understanding of the proposed model. In the description of the system, we used quotation marks for the short name given to each relationship depicted in the figure and bold to identify subsystems. To facilitate the reader, we summarized each subsystem in supplementary material (S1 Table).

The process to build up the model was iterative: literature review, model draft, and discussion with specialists in as many iterations as necessary until the model was considered robust. In this study, this process required 13 steps.

## Boundaries and external influences

**Fig 1:**
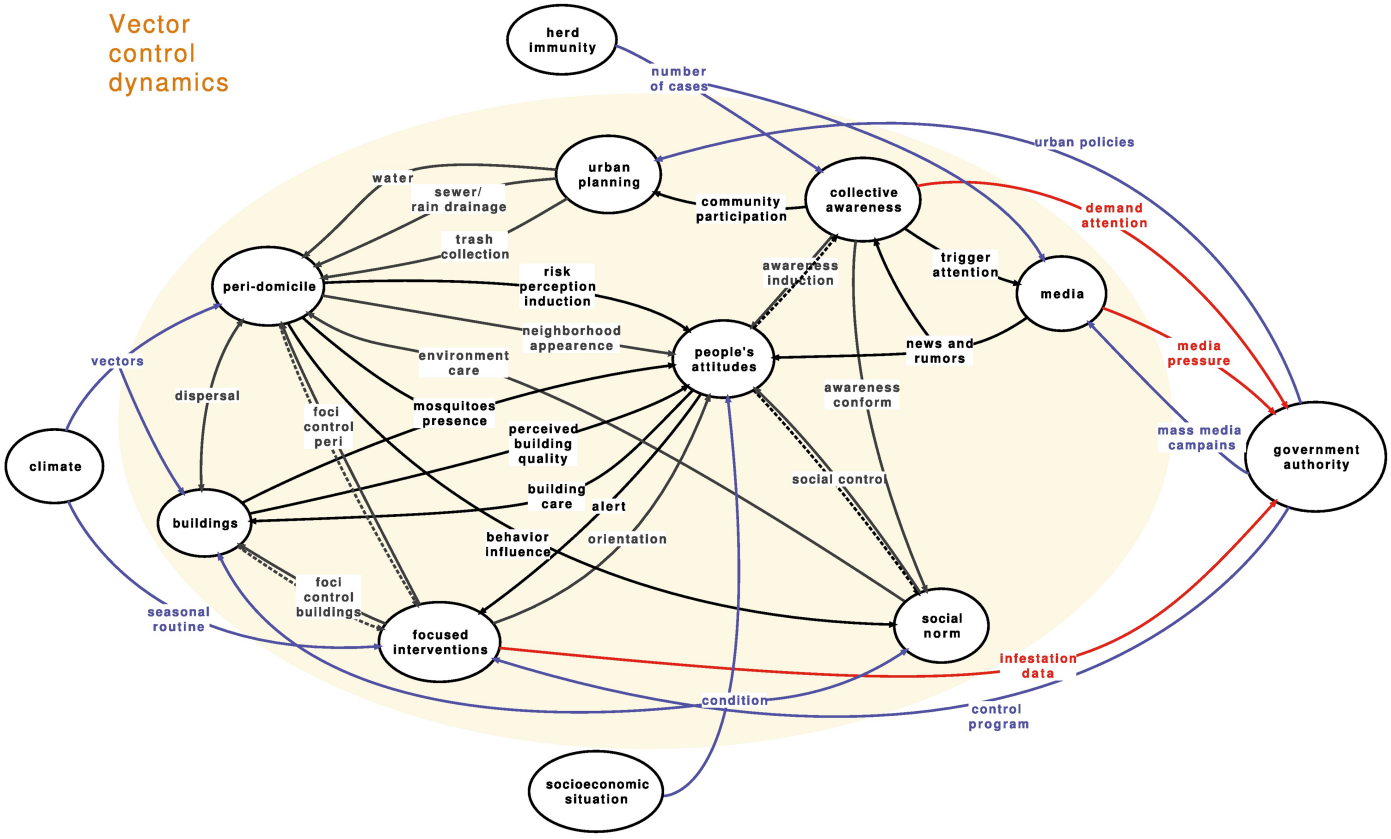
Vector-control dynamics system. Blue and red arrows represent, respectively, inputs from and outputs to elements outside the system boundaries.

Briefly, outside the beige circle that defines the boundaries of the vector-control dynamics system are the main external driving forces, which influence the system through several relationships with internal subsystems (blue arrows) and receive the system outputs (red arrows) (Fig 1). The **government authority**, encompassing national, state, and municipal governments, defines and is in charge of the primary interventions related to *A*. *ægypti* control. Besides, government authorities are responsible for urban planning and allocation of available resources among several priorities, including the public health system.

The **government authority** subsystem represents all coordination levels (national, state, municipal, or other) responsible for defining activities and protocols, including budget, personnel, technical guidelines, approved substances, routines, evaluation, and relationships with other areas of government, such as education and health sectors. The main technical protocols, for instance, indicating which chemical substances should be applied, are defined in general by federal authorities. Some variation on the routines is expected to take into account local aspects. Therefore, the “control program” is the main influence on the day-to-day **focused interventions** for vector control [21].

Another branch of the vector-control strategies, often planned and executed by the Ministry of Health, are the “mass media campaigns,” mostly focused on stimulating individual care of potential breeding sites in households. However, we could not find in the published literature any efficacy assessment of those campaigns and just 1 paper evaluating the mass media communication of dengue surveillance reports, which did not find any association with larvae positive breeding sites in the households [22].

We explicitly included the “urban policies” influencing the **urban planning**, which is administratively independent from the vector-control program but nonetheless has a substantial impact on the mosquito population. The variability of urban infrastructure and autonomy of urban interventions across municipalities, with limited cross information with the local vector-control teams, is relevant [23]. Despite the fact that there are several papers discussing different aspects of urban setting, very few have addressed this issue with regard to vector control [24].

The **climate** is a well-known factor that influences “vector” population controls viral replication within the vector [25], accounting for the seasonality of vector-borne diseases. The temperature above 22–24°C has been associated with *A*. *ægypti* abundance and, consequently, with an increased risk of arboviruses [26]. Droughts, for instance, may impact the number of mosquitoes negatively, decreasing rainfall-sustained breeding sites, or positively, increasing precarious water storage inside households [27]. Strategically, the **focused interventions** on vector breeding sites peak just before the summer (“seasonal routine”), the season with the highest number of expected dengue cases.

**Herd immunity** [28] synthesizes the past experience of the human population with the diseases and the cyclic outbreak pattern: as the number of susceptible people decreases, the probability of an infected person transmitting the virus to a susceptible one (via the vector) decreases as well, until a sufficient naive population accumulates and triggers a new epidemic. The “number of cases” directly affects 2 internal subsystems: **collective awareness,** as disease happens in neighbors or families, and triggers **media** interest.

The last and most important external subsystem is the **socioeconomic situation**, which “conditions” both the **people's attitude**, their neighborhood context (**social norm**), and the household (**buildings**) conditions. The effect of education and income on knowledge regarding *A*. *ægypti* control is well studied [29]. Other aspects potentially affecting the ability for engaging in vector-control activities are, for instance, the high proportion of women (still the gender responsible for housework) no longer exclusively dedicated to this task and trying to include more activities into their workload, especially in the most deprived areas [30].

The main outputs of the system are upon the **government authority**: the influence (“demand attention”) due to the **collective awareness** and the “media pressure”. The main information returned to the government authorities is based on the indicators built on the vector “infestation data” gathered during the usual control activities. Those external relationships regulate and maintain the system, dynamically interacting with the internal subsystems.

## The complex system map for the *A*. *ægypti* population

The system is composed of 8 subsystems. Two of them summarize interventions: **urban planning** and **focused interventions**; 2 are the physical substrate for maintenance of the *A*. *ægypti* population: **buildings** and **peridomicile**; and 4 are constructs dealing with social and psychological aspects. The supplemental material summarizes the main aspects of each subsystem.

The **urban planning** subsystem is responsible for defining priorities, setting the budget, and implementing all urban interventions, including the ones usually associated with mosquito-breeding sites. The aspects included in the model are: water distribution, including regularity, a central issue related to the use of containers that are potential breeding sites [31,32]; “sewerage/rain drainage,” affecting not only the *A*. *ægypti* but *Culex sp*. and the general sanitary quality of the neighborhood [23]; and “trash collection,” the removal of potential breeding sites, such as plastic bottles and other containers, from the streets [33]. These interventions are directed to the **peridomicile**. The **urban planning** subsystem is influenced by the **collective awareness** through “community participation.” **Urban planning** and **focused interventions** are entirely independent of each other, with structurally different work plans and agenda.

The **focused interventions** is the subsystem that encompasses vector control strategies, with 3 main activities: foci control, data collection to estimate vector infestation indices, and population orientation [21]. The first is the elimination of all containers that might be breeding sites and, if elimination is not possible, covering, cleaning, and treating them with larvicides when appropriate. The targets of these activities are the **buildings** (“foci control—buildings”) and **peridomicile** (“foci control—peri”), and include, at the same time, data collection on infestation for the surveillance system (dashed line). The “orientation” on how to keep the household clean of *A*. *ægypti* is supposedly given by environmental health agents during their regular visits to households. The Brazilian routine, in accordance with international recommendations, indicates 6 household inspections each year by trained agents. However, just 7.3% of the households are visited every 2 months, and this number is only slightly higher in areas served by the Family Health Strategy (FHS) [34]. The output link informing the **government authority** on the situation of the control program is based on infestation indices, in spite of limited quantifiable association among vector indices and dengue transmission [35]. Additionally, information—count, type, and size of breeding sites and whether they are active or potential, based on standard specification and observation of *A*. *ægypti* larvae—is an important instrument for evaluating arbovirosis-control programs [21], with just small variation among most countries.

The urban **buildings** are where people live, work, or visit for whatever reason and include buildings that are closed, abandoned, or in construction, either public, commercial, or private. **Buildings** (and **peridomicile**) are where the *A*. *ægypti* feeds on people and lays eggs on available breeding sites. In fact, the domestic *A*. *ægypti* is never found far from human habitation and oviposits in a wide range of manmade containers [36]. Here, we define the **peridomicile** as the surrounding area of each building, roughly defined by the mosquito’s flying capacity, which is influenced by the availability of oviposition sites. Both are the primary target for intervention, due to the anthropophilic behavior of the *A*. *ægypti*. The relevant difference between them is the responsibility for the maintenance. **Buildings** belong to individuals, institutions, or companies. **Peridomicile** is considered a public space with limited, if any, sense of community responsibility [37], particularly in deprived areas. In the case of working spaces, schools, and health facilities, quite often the responsibility is attributed to some impersonal manager, in general, absent [38]. **Buildings** and **peridomicile** exchange *A*. *ægypti* as depicted by the double-headed arrow (“contamination”).

The *A*. *ægypti* females lay eggs preferably in manmade containers (buckets, drums, tires, vases, among others) with clean water and under the shade, among other characteristics that improve survival and growth of their offspring, a behavior that ultimately influences population distribution and abundance [39]. The general aspects of quality of the **buildings** and **peridomicile**, such as internal space, street pavement, running water, and embellishment, are all highly dependent on socioeconomic aspects (“condition”) besides specific characteristics indicating the presence of potential breeding sites, such as water containers not adequately closed, plant pots, and open rain drainage. If lacking essential elements, such as protected water reservoir, sinks, proper bathroom, or adequate rain-drainage system, any orientation or media campaign asking people to eliminate potential breeding sites will not be very successful [40].

The most central subsystem is **people's attitudes** related to the vector-control activities, linked to the other subsystems through 11 relationships. This construct synthesizes a set of feelings, beliefs, and behaviors that are reflected in the practical vector-control activities, basically the “building” care. From the physical subsystems, **people’s attitude** is influenced by the perceived “building quality” and “neighborhood appearance.” The perception of “mosquito presence,” which can be caused by other species, may affect **people's attitudes**, either making all efforts seem useless, as mosquitoes keep biting, or too successful and therefore no longer needed [41]. It is important to observe that the actions that control *A*. *ægypti* population do not affect the more common *Culex sp*., due to differences in vector behavior. The **peridomicile** influences **people's attitudes** as well through the “risk perception induction.” This relationship is dynamic and changes according to sociocultural environment [42] as well as recent outbreaks in the neighborhood or severe cases of the disease in closely related people, potentially modifying **people's attitudes**.

The **social-norm** construct is a synthesis of local beliefs and behaviors, and is slow to change. Trust in the work of the environmental health agents, for instance, is rarely given immediately, especially in the most deprived areas, where a past of neglect often makes government presence not desirable, and carefully designed strategies are needed to allow health workers to access residences [43]. The mutual influence (“social control”) between **people's attitudes** and **social norm** is a positive feedback loop, depicted as a double arrow with 1 arm dashed, as the influence in each direction is not exactly the same nor does it take place at exactly the same time. For instance, throwing trash only in the designated places would decrease the risk of small water containers all over the **peridomicile**. However, such places should be easily accessible, and trash should be collected often enough not to accumulate, in a relationship called “behavior influence.” However, even given this precondition, a change in behavior would be the criticism directed at people throwing trash outside the designated places. In this way, the **social norm** affects the **peridomicile** through a collective “environment care” that may be present in different levels or even absent [44].

Possibly, the most important influence on the breeding capability of a neighborhood is the continuous “building” and “environment care” of the places where people live. Additionally, in a rapidly changing environment (a common factor in most poor neighborhoods), an active participation of the community through providing information to individuals in charge of the **focused interventions** (“alert”) on all breeding sites—closed or in-use buildings and peridomicile—is very important. However, in most cities, this participation is limited to a central telephone number [45], discouraging this contact.

Taken collectively, a general public feeling, here denominated **collective awareness**, is important for all transmissible disease [46]. In the case of mosquito-borne diseases, we hypothesize that **collective awareness** influences the individual attitudes in a positive immediate feedback loop (“awareness induction”) and the **social norm** (“awareness conform”).

Finally, **media**, both traditional (television, radio, newspapers, etc.) and the internet, spreads information (“news and rumors”) that influences **people’s attitude** and **collective awareness**, the latter triggering the media’s attention (“trigger attention”). **Media** is also used by the government authorities in “mass media campaigns” [47].

To decrease the total amount of mosquitoes is the ultimate objective of any vector-control program. **Buildings** and **peridomicile** are where mosquitoes breed and feed on people. The government mainly intervenes through the direct action of environmental health agents, which is intermittent and scheduled according to routine protocols and at some moments to the government’s sensitivity to public awareness. Individual care is called upon through mass media campaigns and the not-so-regular home visits. Thus, the question is which interventions can help change **people's attitudes** towards eliminating breeding sites and maintaining care over time. Next, we will evaluate the feedback loops to better understand the main forces keeping the system as it is: sufficient mosquito population to keep the diseases endemic.

### Feedback loops

**Fig 2:**
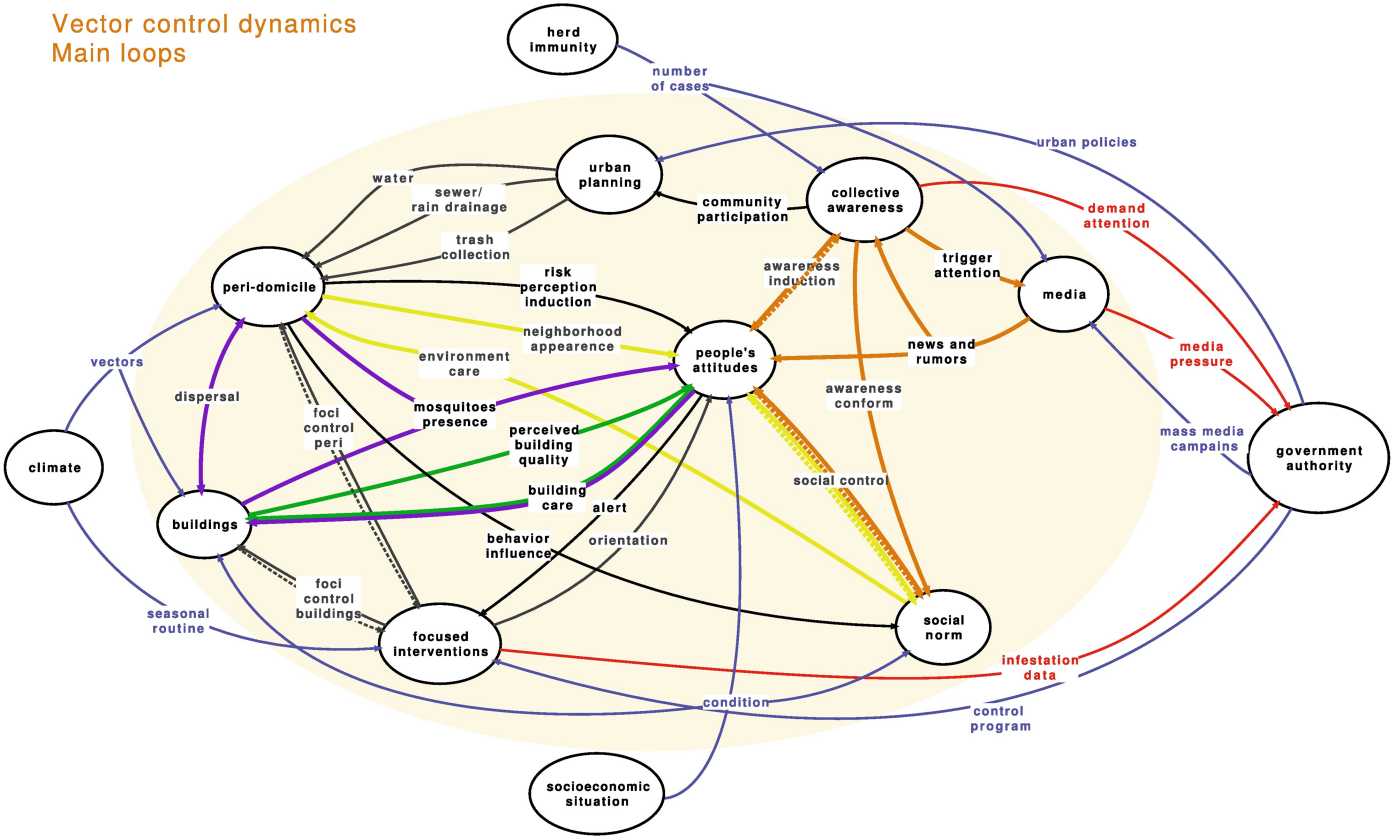
Investigated feedback loops. Blue and red arrows represent, respectively, inputs from and outputs to elements outside the system boundaries. Other colors map to feedback loops under analysis.

The model’s final objective is to support the choices of actions that may change the vector infestation. In this section, we will therefore present and discuss its main stabilizing feedback loops (Fig 2). Eve, n considering that these loops interact with each other, we can focus on each one per se, helping to understand the model as a whole.

Focusing initially on the relationships to and from the **buildings**, the first obvious loop (in green) is a positive feedback: better “perceived building quality” returns more “building care” neither linearly nor always. Building care is simply easier if, for instance, there is regular water distribution and containers are adequately protected. As the quality of the household is not easily changed, this loop fluctuates mainly due to changes in the **people's attitudes**.

Another loop (in purple) between **buildings** and **people's attitudes** derives from “mosquito presence,” a relationship affecting **people's attitudes** with inputs from both the **buildings** and **peridomicile**. The perception of mosquito presence is not specific for just *A*. *ægypti* and may influence the maintenance of “building care.” If no decrease in the mosquito presence is perceived, as may be the case in most areas due to a favorable environment for the *Culex sp*., the attitude towards “building care” can be a feeling of uselessness. Again, changing the environment to control for other mosquitoes species, such as the closure of open sewerage and the cleaning of canals, is a long-term sanitation proposal and not completely viable. The number of mosquitoes may decrease, but depending on the climate and local conditions, mosquitoes are part of the environment. Additionally, the spread of *A*. *ægypti* from 1 **building** to another and to the **peridomicile** (“contamination”) is a powerful relationship for maintaining the stability of the system: just a few buildings not in pristine condition are enough to (re)infest the whole area, creating and spreading a feeling of futility, and thus affecting the long-term sustainability of the strategy.

Attitudes and behaviors of each person are not independent of the group opinions and a **social norm** emerges from several inputs. Although slow to change, a few examples related to health behaviors did happen, such as the decrease in tobacco consumption in several countries despite the need to keep improving legislation [48]. However, all examples relate to public spaces, not to private households. A positive loop (in orange) towards changing the **social norm** related to *A*. *ægypti* control would slowly improve the environment (“environment care”) and positively stimulate **people's attitudes** (“neighborhood conditions”). Additionally, at least in public behavior, such as with garbage disposal, “social control” is expected to create a new conformity rule, in our case, less favorable to mosquito-breeding sites. However, the role of private **buildings** and their potential to contaminate the neighboring buildings cannot be overlooked. The combination of those 3 loops tends to stabilize the whole system.

In situations in which **collective awareness** increases (in brown) either during an epidemic or as a result of an intensive “mass media campaign,” a positive reaction may happen, leading to a control of breeding sites, as desired. Again, this does not include every household, nor does it occur for a long time. Especially if the number of cases decreases, the media interest and the public sector commitment wane.

In general, the feedback loops we have focused on here are more immediate without a large time lag between changing 1 element and the cascade of events, with the exception given to changes in the **social norm**. Several long-term influences are depicted in the model, originating in the urban interventions and social norm. However, the first moves so slowly that, in fact, it could be left outside of the proposed system, as an external influence. Our objective in bringing it inside the system was to raise awareness of its importance, even in the short term. If building an adequate water supply, sewerage system, and rain drainage are beyond the scope of the *A*. *ægypti* control, and in fact are associated with several other health problems, trash collection can be improved much more quickly in underprivileged communities, with noticeable impact. Interventions on the relationships and feedback loops highlighted above give faster results and are the usual approach of the **government authority**. Good results occur in the short term but are hard to maintain in the long term, exactly due to the resistance to change found in **people’s attitudes** and **social norm**.

The **focused interventions** subsystem is the privileged space for vector-control interventions. It presents few influential relationships, as the program is organized as a routine protocol with little external influence. **Collective awareness**, for instance, only affects the interventions through the **government authority**, in a very long pathway from the **people's attitudes**, **social norm**, or even the **media**. There is very little potential for a fast response due to, for instance, an increase in the number of cases of any of the diseases transmitted by the *A*. *ægypti*. It should be noted that this lack of timely response to the **people's attitudes** is discouraging. The investment of people's time and energy for many years in an unpleasant, albeit necessary action, without any immediate reward, just to prevent a future event that has a low probability of happening, is a key aspect we need to address better. New or transformed relationships should be established so that a short feedback loop could modulate the focused interventions, not only in emergency crisis as an extemporaneous action.

## Using the proposed model to discuss an intervention

**Fig 3:**
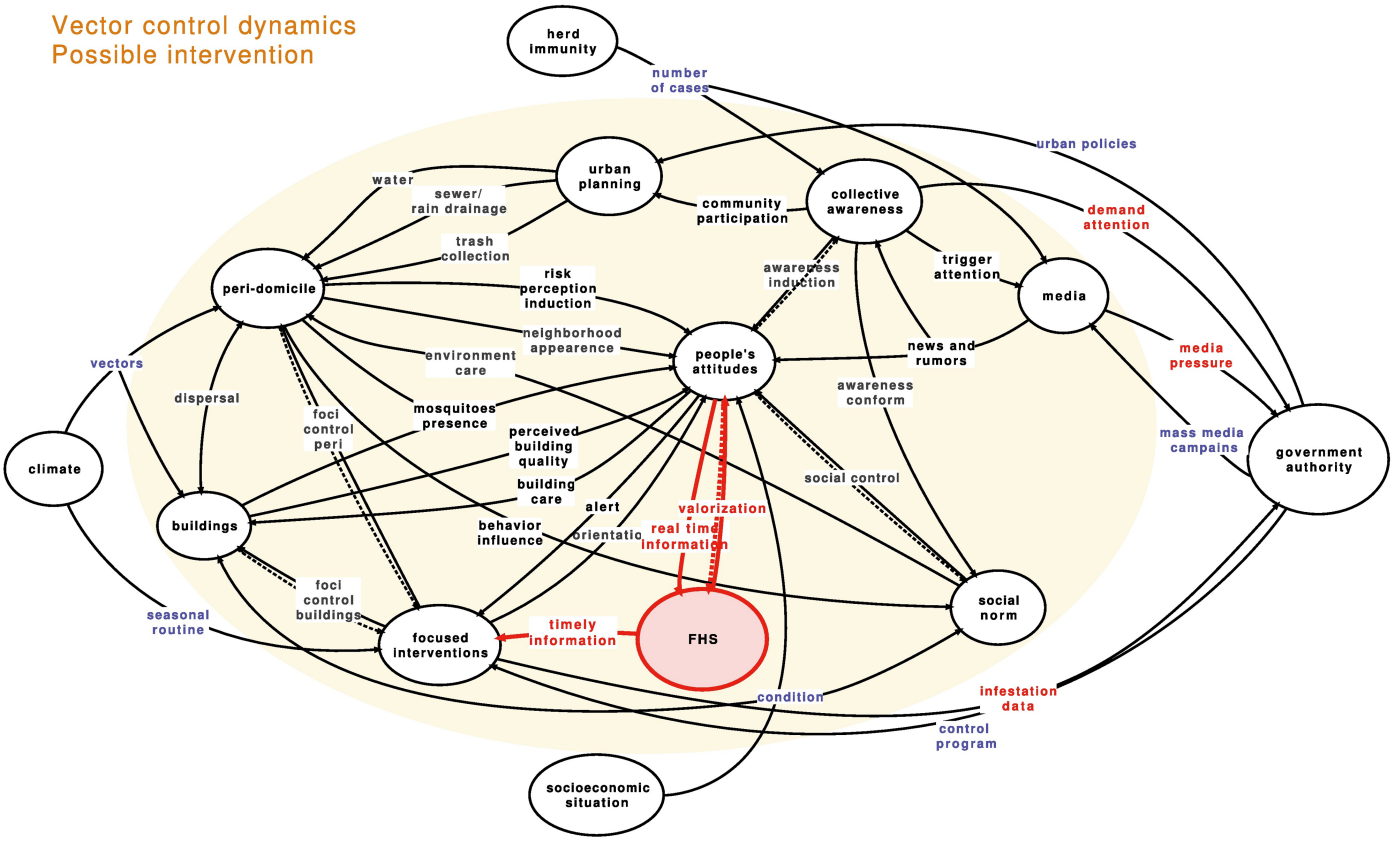
Proposed inclusion of the Family Health Strategy (FHS) into the vector-control dynamics system. Red arrows and node represent the inclusion of the FHS as a new intervenient subsystem.

A possible intervention on the system could be the inclusion of 1 more subsystem in the vector-control dynamics system: the primary health care structure, organized in Brazil via the Family Health Strategy (**FHS**) (Fig 3). The FHS covers 130 million people, the poorest 66.5% of the Brazilian population, with 41,167 family health teams working and 271,524 community health workers hired in the program [49]. In spite of recommendations that information be exchanged between both local health agents, family health and environmental health agents, no practical orientation exists.

The family health agents visit each household in their areas once a month, in general. Additionally, the FHS team includes a doctor and a nurse who assist the population of the area and are responsible for the notification of diseases. Therefore, they are the first to come into contact with complaints and disease cases, even among those who prefer not to go to a medical appointment. From this point, if a link could be established between agents, the information would be available to the local team of environmental health agents very quickly. An immediate response to any condition—increase in the number of cases of diseases of interest, complaint about mosquitoes or about breeding sites in the neighborhood—could put the whole system into another frame.

The **FHS** in this proposal would link to **people's attitudes**, giving adequate “valorization” to complaints and general feelings considered as “real-time information” and stimulating individuals’ participation in control activities, particularly reporting potential breeding sites. From there, a link should be established to the local technical people (“timely information”) to address each and all complaints, especially those related to potential breeding sites. The main activities of the vector-control program do not change. The change is in the response to the population’s participation and the role of primary health care. Individuals’ perception of participating may improve the care directed to potential breeding sites.

Just 3 relationships were included in the proposal, but the influence would spread through the others. The family health agents have free access to most households, especially the most deprived. Even in places where the resident does not accept his presence, knowledge about potential breeding sites would be more readily available, stimulating a dialogue to facilitate intervention (“foci control—buildings”). The risks of failure are evident, as any change in the usual routines for health agents is not easily done. Other tools could be included to stimulate individuals’ participation, such as activities in schools or the use of social media. However, the sustainability of any action is only guaranteed if incorporated into the day-to-day routine of the vector-control program.

The proposed solution is not entirely new, and it might be considered somehow evident. In 2009, a folder with basic information about dengue [50] was distributed to all family health agents, saying, “Our call is for you to share the information in this booklet on how to avoid the disease with your community, creating partnerships with institutions—neighborhood associations, churches, schools, merchants, local trash collection companies, and others who can help to build up a better space where they live and work” (authors’ translation). In the last pages of the document, environmental health agents are cited, but no specifications on the work of each type of agent are provided. No proposal of joint work or of a timely response is available in the document. The lags between detection of a potential breeding site or increase in the number of cases and intervention by the vector-control program are not perceived.

What is initially needed and is probably the most relevant action for our purposes is an integration among all local government agents, beyond the usual platitudes. The reason this does not already exist is possibly that different agencies jealously guard their expertise and avoid being pushed to do anything that is not formally among their attributions. To be able to get a deeper understanding on how to surmount this problem, some research opening up both the **FHS** and **focused intervention** subsystem is needed. On the other hand, just the participation of the health family agents does not guarantee changes in **people's attitudes**. Timely and reliable response to any positive change in **people's attitudes** is essential to counterbalance the general distrust of government agents, which could impair the desired effect. More investigation on motivation, risk perception, life perspectives, and empowerment should be carried out.

## Conclusions

Complex systems modeling is necessarily multidisciplinary and even transdisciplinary, not just superimposing the knowledge of several people from different backgrounds but integrating it creatively. In addition to the experience accumulated in the Brazilian dengue control program, we incorporated in this paper several interactions with entomologists, environmentalists, geographers, climatologists, sociologists, and anthropologists from other countries as well. Especially important was the inclusion of professionals working in the primary health care system both on the front end, supervising the family health agents, and managers of the family clinics, responsible for local planning.

One question immediately arises: is the proposal valid beyond the areas in Brazil where the FHS is available? No, it is not. As expected in any complex situation, this is not a generic solution that can be exported to other countries, even with similar epidemiological and socioeconomic profiles. It is highly dependent on the actual environmental factors (in the systemic sense, not just the physical environment). Even in Brazil, other strategies should be devised to deal with middle-class neighborhoods, where the FHS and visits of environmental health agents are not well accepted, as studies have shown that more than 70% of the residents in affluent areas are seropositive for dengue fever [51].

Quoting a recently published book on system science: “Both population scientists and policymakers assume these interventions have direct, linear effects […] that are consistent across different places in different times […]. The trouble, however, is that interventions founded on simplifying a complex world often do not work” [52]. The simple and generic solution did work in the 1960s, based on a vertical program and an environmentally aggressive substance. We need to expand the approach developed here to as many places as possible in order to find reliable and sustainable solutions. The development of a conceptual model is an indispensable aspect to deal with complex health problems, and this approach has implications as well for future research [53].

This article makes 2 substantial contributions, namely, the methodological approach and the general aspects of the proposed system to deal with the long-term sustainability of *A*. *ægypti* control. Despite the fast response to risk in areas where there were previously no mosquito-transmitted Zika cases, aerial insecticide spraying [54] is not sustainable in the long term. Resistance to the products is expected and other environmental damages as well, such as decrease in pollinators populations, for instance, bees [55]. The problem here is uncertainty over how long it will be cost-effective and how to maintain the mosquito population below the epidemic threshold in the long term. In fact, the greatest challenge for any given complex problem is not the short-term changes but influencing the system towards a new desired dynamic equilibrium.

To the best of our knowledge, this is the first paper using complex systems approach to analyze a vector-borne disease. Interestingly, a recent review on arbovirosis vector control concluded by stating the “remarkable paucity of reliable evidence for the effectiveness of any dengue vector control method” [56]. Why is it so? Our hypothesis is that mosquito control is still a practice derived from mainly just 1 discipline: entomology. Most knowledge is laboratory driven. Human sciences knowledge is still just a secondary tool to make people behave as they should with regard to mosquito control. Despite thousands of papers published on *A*. *ægypti* control, knowledge gaps were found while building the model. This is another gain from an integrated approach.

The proposed model should be further developed, both opening up each of the subsystems and evaluating parts of the feedback loops using simulation techniques. Agent-based and system-dynamics models and empirical studies could be devised in order to explore how to change **people's attitude** in a bottom-up, self-organizing system. However, from the point of view of developing the current proposal, only a thorough, qualitative, soft system approach would integrate enough knowledge from several different areas to build up the model.

This paper was only possible due to a long-term interaction with entomologists, epidemiologists, health services researchers, and a reasonable knowledge of the FHS in one of the most challenging cities in Brazil, Rio de Janeiro. We hope that the approach undertaken here brings new insights to the field, which could be useful for protecting the population.

## Supporting information

S1 Table Brief description of the subsystems composing the system. (PDF)
