# Supplementary material for: Aedes ægypti control in urban areas: A systemic approach to a complex dynamic
Source: PLoS Negl Trop Dis. 2017 Jul 27;11(7):e0005632. doi: 10.1371/journal.pntd.0005632 (PMC5531422; doi:10.1371/journal.pntd.0005632)
Supplement: S1 Table — (PDF) [file pntd.0005632.s001.pdf]

Brief description of the subsystems composing the system

**External subsystems**	
Climate	Rainfall, temperature, and humidity, main climate aspects that condition mosquito reproduction, survival, activity
Heard immunity	Population immunity level, which, if below certain threshold, increases the probability of outbreaks
Government authority	All government coordination levels (national, state, municipal or other) responsible for defining activities and protocols, including budget, personnel, technical guidelines, approved substances, routines, evaluation and relationship with other areas of government, such as education and health sectors.
Socioeconomic situation	Subsystem combining environmental conditions for mosquito proliferation and socio-cultural aspects related to attitudes
**Internal Subsystems**	
Urban planning	All urban interventions, related directly or indirectly to potential mosquito breeding sites, through water distribution, sewerage system and trash collection
Focused intervention	Vector control strategies for households and peri-domicile, including removal of potential breeding sites, data collection on the vector and population orientation
Peri-domicile	All public spaces, including streets, squares, commuting areas
Buildings	Private and public buildings,such as schools, shops, health services, etc
Social norm	A synthesis of locally accepted beliefs and behaviors related to potential breeding sites, such as trash deposits, individual household care
Media	Both traditional media (TV, radio, and newspapers) and the Internet
Collective awareness	General public feeling about the importance of vector control
Peoples’ attitude	A construct synthesizing a set of feelings, beliefs, and behaviors that are reflected in the practical vector control activities
